# Dynamics of Team-Based Learning in Molecular Biology: Insights and Reflections From Undergraduate Medical Students

**DOI:** 10.7759/cureus.76180

**Published:** 2024-12-22

**Authors:** Anila Jaleel, Umair Aziz, Shahila Jaleel, Ghulam Farid, Zahid Bashir, Rana Muhammad Hassaan Sikander

**Affiliations:** 1 Biochemistry, Shalamar Medical and Dental College, Lahore, PAK; 2 Pathology and Laboratory Medicine, Shaikh Khalifa Bin Zayed al-Nahyan Medical and Dental College, Lahore, PAK; 3 Library, Shalamar Medical and Dental College, Lahore, PAK; 4 Library, Shalamar Hospital, Lahore, PAK; 5 Forensic Medicine and Toxicology, Shalamar Institute of Health Sciences, Lahore, PAK; 6 Medical Education, Shalamar Medical and Dental College, Lahore, PAK

**Keywords:** collaborative learning, communication skills, conceptualization, medical school, teamwork

## Abstract

Objective: To investigate the dynamics of collaborative learning in team-based learning (TBL) through students’ reflections and feedback.

Methods: A phenomenological mixed-methods approach was adopted where the survey and reflections were conducted concurrently after the TBL session and the results were analyzed. The study employed a mini-cluster technique to include all first-year MBBS students of batch 2023-24 with an age range between 19 and 22 years. Qualitative and quantitative data were collected from feedback and reflections submitted by 107 first-year MBBS students at the end of the TBL session.

Results: The study found five main themes related to students’ engagement with the session, i.e., (1) conceptual clarity as the foundation, (2) collaborative learning culture, (3) communication skills, (4) informative and useful team discussions, and (5) interactive learning for team development. The majority of the respondents, 93 (87%), listened to classmates and coordinated with group members in discussion; 77 (72%) were willing to learn from other group members and contribute ideas to them; 79 (74%) came prepared for the session; and 75 (70%) found learning material assigned to them as appropriate. Respondents had positive perceptions regarding their participation in group work and were strongly inclined to agree with the statements provided.

Conclusion: The study highlights the positive effects of TBL in promoting peer-to-peer engagement, active engagement, teamwork skills, and knowledge sharing among undergraduate medical students, ultimately contributing to an effective learning experience.

## Introduction

Team-based learning (TBL) is a collaborative learning approach that has gained popularity in recent years due to its effectiveness in promoting active learning, critical thinking, and problem-solving skills [1,2]. It is a structured learning method that involves students working in small groups, requiring pre-class preparation by students and in-class application of knowledge to solve complex problems, discuss case studies, and apply their knowledge to real-world scenarios. It originated as a teaching method in business schools in the early 1990s and was developed by Dr. Michaelson [3,4]. It is an effective teaching method across a range of disciplines, including medicine, nursing, engineering, and business [5,6]. It has been associated with increased student motivation, satisfaction, and knowledge retention, highlighting its positive impact on the overall learning experience [7-9]. TBL provides opportunities to use and enhance conceptual knowledge through a variety of processes, such as preparation, readiness assurance testing, feedback, and the application of knowledge through clinical problem-solving activities [10,11]. The TBL's simplified framework tackles resource constraints that are frequently faced by universities. Enabling a large number of students to participate in small group learning with a limited number of knowledgeable facilitators is another important benefit of TBL [12,13]. There is a scarcity of literature on the conduction of team-based learning in developing countries, as the majority of studies tend to concentrate on its application in more developed regions [14]. Therefore, this study explored the dynamics of collaborative learning in TBL and examined the perception of TBL by undergraduate medical students in Pakistan. The study aims to contribute to the growing body of research on TBL and to provide insights into the dynamics of collaborative learning in medical education. The objective of the study was to investigate the dynamics of collaborative learning in TBL through students’ feedback and reflections.

## Materials and methods

This study was carried out among 107 medical students from June 2024 to August 2024 at Shalamar Medical College, a private medical college in Lahore. A phenomenological mixed-methods approach was adopted where the survey and reflections were conducted concurrently after the TBL session, and the results were analyzed. The study employed a mini-cluster technique to include all first-year MBBS students of batch 2023-24 with an age range between 19 and 22 years. Students who were absent on the day of the session or did not consent to participate were excluded from the study. Students were invited to participate in the study by distributing consent forms at the beginning of the study. Those who were willing to participate were given a feedback questionnaire and were asked to write reflections about it. Students were provided with information about the purpose of the study to facilitate informed decisions about participation. It was emphasized that participation was entirely voluntary and that students had all the right to withdraw at any stage of the study and would not be penalized. Personally identifiable information, such as names, was not collected. Confidentiality and anonymity were assured. The study was granted an exemption from the ethical review board of the college (IRB No. 0744/SMDC-IRB/AL/2024-041).

The TBL session was conducted for two hours with all the steps of the session being followed. During the preparatory phase, students were given the session’s learning objectives as well as clinical cases linked to molecular biology through the Learning Moodle System (LMS) to study independently prior to the TBL session. They were asked to revise the topics covered in previous lectures in preparation for the TBL session. In-class sessions include an Individual Readiness Assurance Test (iRAT), in which students were given a pre-test at the start of the session consisting of 10 multiple-choice questions (MCQs) linked to the pre-assigned material to assess their individual readiness.

Following the RAP, the class was divided into four groups (e.g., Groups A, B, C, D) for the TBL session, and group representatives were identified in each group. These groups were further subdivided into small groups of 5-6 students in each group and engaged in discussion of case studies provided to them for preparation. They worked in their teams to analyze the problem, discuss potential solutions, and apply their knowledge to solve it. The facilitator was there to encourage active participation and discussion among students.

A post-test was conducted to assess the readiness of participants after collaborative study in groups and comprised of 10 to 15 MCQs, which was called the Team Readiness Assurance Test (tRAT). Objective assessments were conducted to measure any changes in academic performance after individual study (pre-test) and after collaborative study in groups (post-test). These are followed by reporting and discussion, during which each team presents their solutions or findings to the class. This stage encourages peer teaching and allows for comparison and discussion of different approaches. Instructors also provided additional insights, clarified concepts, or addressed misconceptions during this phase. Group representatives presented their findings and explanations to the class, fostering peer-to-peer learning and knowledge sharing. The session focused on topics such as DNA replication, transcription, and translation in biochemistry. Students were evaluated based on their participation, understanding of the cases, and the accuracy of their explanations. Marks were awarded to teams (A, B, C, D) based on the quality of their responses and contributions during the TBL session. The following case scenarios were given to the students. Feedback was taken from the students on the questionnaire, and they were asked to write reflections in 15 minutes.

The data was extracted from a structured feedback questionnaire distributed to first-year MBBS students regarding perceptions of TBL, based on a three-level Likert scale after the TBL session. The students were encouraged to write their reflections and responses based on their TBL experience and collaborative group discussions to delineate their level of satisfaction. Qualitative data was collected from their open-ended reflective writings, and themes were identified using NVIVO by creating codes and sub-codes. Quantitative data was analyzed using IBM Corp. Released 2015. IBM SPSS Statistics for Windows, Version 23.0. Armonk, NY: IBM Corp. Frequency and percentage were determined for items on the Likert scale. Qualitative data was analyzed using NVIVO. To identify themes, code themes, and subthemes. Thematic analysis was done for qualitative data using a phenomenological framework. Each reflection was read by members of the team, and impressions were discussed. Codes and sub-codes were identified, and coding disagreements were resolved by consensus. The study team discussed the coded data to identify themes related to the objectives of the study.

Analysis of qualitative and quantitative data was initially done separately. The major themes were then considered alongside the survey findings. Qualitative and quantitative data were aligned by analyzing themes identified along with the findings of the questionnaire. The study members analyzed the areas of convergence and divergence and comprehended the findings in the questionnaire to have detailed discussions in reflections.

## Results

**Figure 1:**
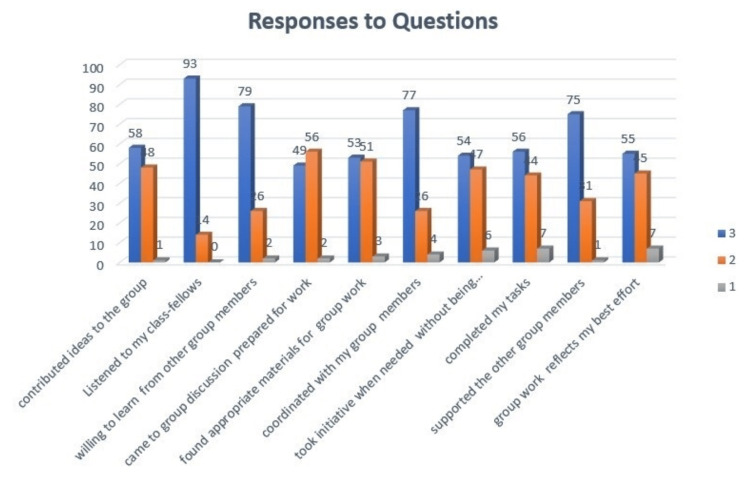
Quantitative analysis of feedback by students

A total of 107 participants were surveyed using a structured questionnaire with a Likert scale where 1 denoted ‘Disagree,’ 2 denoted ‘Somewhat Agree,’ and 3 denoted ‘Strongly Agree.’ Participants were asked to choose a response that best represented their opinion on the given statements. The responses of the participants were plotted on a clustered column chart (Figure 1).

The majority of the respondents, 93 (87%), strongly agreed that they listened to classmates and coordinated with group members in discussions. Others, 77 (72%), strongly agreed that they were willing to learn from other group members and contribute ideas to the group, 79 (74%). They came prepared for session 75 (70%), found learning material assigned to them as appropriate for group work 58 (54%), and took the initiative to participate when needed 53 (49%). They completed the tasks on time (54%), supported other group members (56, 52%), and felt that their group work reflected their best effort (55, 51%). The rest somewhat agreed, and only 3% of respondents disagreed with the statements (Figure 1). Table 1 shows clinical cases given to students prior to the TBL session for individual study. 

**Table 1:** Clinical cases of molecular biology The table shows clinical cases given to students prior to the TBL session for individual study.

Case No.	Description
Case 1	A 23-year-old diabetic woman exhibits symptoms of dysuria, chills, and a high-grade fever. Urinary analysis reveals germs in the urine, and physical examination shows costovertebral pain. The doctor starts a 5-day treatment of ciprofloxacin, suspecting a complex UTI.
	What is the mechanism of action of ciprofloxacin?
	How does ciprofloxacin prevent the proliferation of infection?
Case 2	A 34-year-old male of Italian descent undergoes an annual physical checkup. Despite being in good health, he has mild anemia and a family history of anemia. The doctor suspects thalassemia minor, which is often caused by alterations in RNA splicing.
	What is the mechanism of RNA splicing?
	In which process does RNA splicing occur?
	What alteration in RNA splicing can lead to thalassemia?
Case 3	A 54-year-old man presents with a low-grade fever and ineffective cough for 3–4 weeks. The physician suspects atypical pneumonia caused by Mycoplasma pneumoniae and prescribes erythromycin as empirical treatment.
	What is the mechanism of action of erythromycin?
	How does erythromycin inhibit bacterial growth?
Case 4	A new patient fears colon cancer due to a family history of right-sided colon cancer. Several relatives developed the cancer with visible polyps only on the affected side, all diagnosed before the age of 45.
	What is the reason for the development of this type of colon cancer in families?
Case 5	A patient presents with cramps, vomiting, and chills after eating wild mushrooms he picked earlier in the day.
	Why does the patient develop symptoms after eating mushrooms?
	What is the mechanism by which these symptoms develop?
Case 6	A 2-year-old girl complains of a tight neck and high-grade fever. Physical examination reveals petechiae and positive Brudzinski and Kernig signs. The pediatrician suspects meningitis and prescribes a medication that inhibits the formation of prokaryotic peptide bonds.
	What is the drug prescribed and how does it act?
Case 7	A 2-year-old boy with an ear infection is treated with amoxicillin, but the infection does not resolve. After switching to azithromycin, the infection is cleared, and lab tests reveal the bacterium was resistant to amoxicillin.
	What is the mechanism that leads to bacterial resistance in patients?

**Figure 2:**
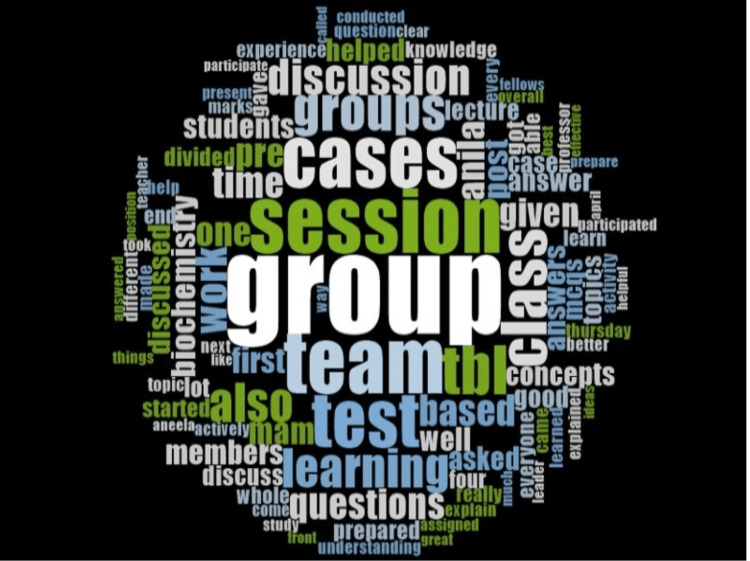
Word cloud shows theme and subthemes identified through students' reflections

Group, discussion, session, cases, team, TBL, learning, concepts, and questions were the most frequently coded words (Figure 2).

**Figure 3:**
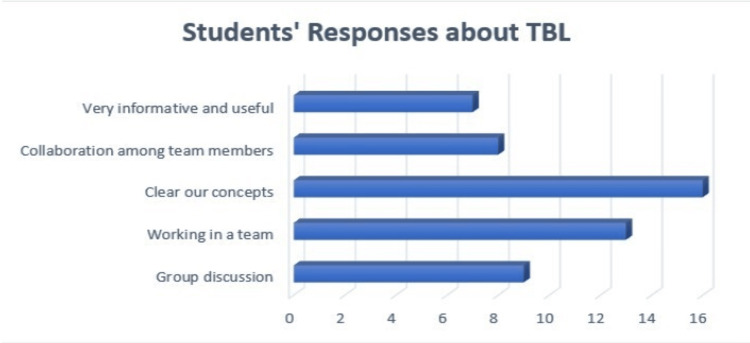
Color-coded themes identified from reflections

The occurrences were counted and plotted on a ‘Clustered Bar Chart’ to gain a deeper understanding of the data (Figure 3).

The themes identified were conceptual clarity as a foundation, collaborative learning culture, communication skills, very informative and useful team discussions, and interactive learning for team development (Table 2).

**Table 2:** Themes and subthemes identified in the study

Themes	Subthemes
Conceptual Clarity as Foundation	Concept
Collaborative learning culture	Collaboration
Communication skills as glue	Communication
Informative and useful team discussion	Team Discussion
Interactive Learning for Team Development	Interactive learning

The students express gratitude for the session, emphasizing its helpfulness in clearing concepts and aiding memorization. It helps them to grasp the concepts clearly and increases interest in the TBL session itself, as evident from the quotations of participants. The strategy of dividing students into groups for questioning added an interactive dimension, promoting interactive learning and conceptual understanding.

Collaborative learning is crucial for effective teamwork, promoting a culture of mutual learning. Students actively assist peers in understanding challenging concepts, demonstrating the productivity of discussing problems and acquiring new knowledge during sessions. Hindrances in collaboration were a lack of confidence and ill-preparation as mentioned by the participants in Table 3.

**Table 3:** Participant’s quotations against the themes identified The table shows the quotations of students for different themes identified from their reflections. It shows their personal views about the team-based Learning (TBL).

Themes	Quotations
Conceptual clarity as the foundation	“I thought this session was helpful because it helped us clear our concepts and memorize most of the topics”. (Participant 6)
	“We cleared our concepts regarding every topic. There was a great discipline in the class. It was a new experience for me but was a great one”. (Participant 19)
	“I am happy with myself for helping my group mate out with revising the process of gene splicing. It felt nice to help them out and it solidified my own concept as well”. (Participant 21)
	“In this lecture the teacher divided us into groups and started asking questions group wise. In my opinion it is very effective method as we get to understand the concepts from different people in different ways. Such activities help us to study in lighter environment.” (Participant 30)
	“Our concepts got more cleared due to TBL and we learned to cooperate with other fellows. After discussion Professor took a post assessment test and I personally felt that I can give answers more clearly now. I will anxiously wait for next TBL session”. (Participant 31)
	“I was ecstatic to hear that our Group C had secured the first position. At the end we had a post discussion test that we quickly solved, but this time all my answers were correct and it took me less time to answer them since I was having no confusion”. (Participant 34)
	“I really enjoyed this way of learning since it consolidated all my concepts with the constant repetition. However, when the professor asked me a question, I got anxious and forgot the answer even though I knew it, that really set me off”. (Participant 50)
	“Helping others also deepens your own understanding of the concepts”. (Participant 65).
Collaborative Learning Culture	“I found that my weakness is lack of confidence to answer the question in front of everyone. I'll try my best to overcome my weaknesses”. (Participant 2)
	“We were upset because we couldn’t get the first, but we appreciated all of our members because we really had a great time in this session”. (Participant 5)
	“With the help of teamwork, I will be able to become a good doctor capable of listening to others’ advice and help others”. (Participant 9)
	“All the groups had a wonderful collaboration in them. I, along with other members, also contributed ideas to the group. I listened to other class fellows and learned from their ideas”. (Participant 10)
	“My preparation was below par compared to my teammates; thus, I was not able to contribute to the maximum of my ability”. (Participant 13)
	“Discussing the problems with my fellows and sharing my thoughts on it was productive...such sessions should be conducted back-to-back in our class.” (Participant 69)
	“My greatest strength during group work: I helped my peers in understanding tricky concepts and tried to answer the questions asked by the facilitator”. (Participant 103)
Communication Skills as the Glue	“Teamwork was the best initiative taken by Shalamar Medical and Dental College as it helped us learning new things and developed a sense of good communication skills with our members”. (Participant 12)
	“Where there are strengths, there are weaknesses. My greatest weakness was that of miscommunication. This is highly attributed to my fast speech. I speak very fast, and this leads to some words being unclear. My teammates were having a bit of difficulty keeping up with the pace, so I had to repeat my statements once or twice”. (Participant 22)
	“I tried to be the best version of myself. My only weakness is lack of confidence, and I am trying to cope with this difficulty. Inshallah I will overcome this weakness”. (Participant 32)
	“The teacher asked me to elaborate the case 1. I explained the case and got cheers from my group and teachers. At the end of the lecture, the teacher announced the results, and my group won the TBL test”. (Participant 37)
	“The best thing about this lecture was that I was the team leader and was able to participate and present ideas”. (Participant 46)
	“During the class discussion…the concepts were explained in such a way that I was able to absorb the new information easily”. (Participant 48)
Informative and useful Team Discussions	“In my opinion, this session was very informative. Ma’am helped a lot to understand difficult things. The other staff members were also cooperative. At the end I have to say that these types of sessions are so useful”. (Participant 8)
	“Overall, it was an amazing experience which helped us a lot in learning and understanding”. (Participant 17)
	“Each and every student was eager to learn something new and which happened also, overall, it was a good practice”. (Participant 28)
	“It was really good and very fun and interesting. My God really have too much mercy on me. Everything in which I present gets victory”. (Participant 33)
	“I need to be more creative next time in order to contribute better ideas. This teamwork enabled me to ponder over my weaknesses. Now I will surely work on my deficiencies and will become a better team member”. (Participant 42)
	“It's important to establish clear goals and roles, communicate effectively, and respect each other's contributions. When done right, teamwork can lead to increased productivity, creativity, and satisfaction for everyone involved”. (Participant 43)
	“Before taking the TBL session I thought that it will be really a hectic lecture. But when the lecture started, I was wrong...Because Professor made it so easy that it was really informative as well as really good”. (Participant 45)
	“The session was very interactive. It was very informative too. The whole session was basically about learning to interact to increase your knowledge and understanding”. (Participant 76)
Interactive Learning for Team Development	“I contributed by telling my team members what to say, however, I was too shy to actually go to the front and present”. (Participant 1)
	“Working in a team can also help to improve your communication and develop a sense of camaraderie…overall team work can be incredibly rewarding experience”. (Participant 3)
	“Working in a team has been something I have liked doing since my A’ levels. Learning in small groups helps you memorize stuff better, that is what I took away from this TBL session”. (Participant 15)
	“You can work as a team to move a couch up a flight of stairs, launch a work project, or play soccer. Defining teamwork is simple, but understanding how to work well as a team can be complicated”. (Participant 16)
	“The Team Based Learning session was an interactive learning session…I personally benefited from this session because the ambiguities I had regarding a few topics were cleared by the end of this session”. (Participant 20)
	“Usually when you learn 5 to 6 topics individually, it takes a lot of time but when you're doing it together, you can save a lot of time. So, this way this session helped us a lot”. (Participant 36)
	“We also learned to work as a team. This will help us in our professional lives, as a doctor alone, cannot make great things to happen”. (Participant 87)
	“Overall, the team-based learning session was a positive experience. It allowed me to work with my peers, gain new insights into the topic, and feel a sense of accomplishment as a team”. (Participant 102)

Effective communication is crucial for team collaboration and successful outcomes, as it binds team members together and converts conceptual clarity into actionable plans. Participants candidly recognized their weaknesses in communication as elaborated. The TBL session acted as a valuable opportunity to interact with peers, increase knowledge, and deepen understanding of the subject matter. Learning new things was the core idea of this session. Teamwork also enabled the students to observe their weaknesses as evidenced by their perceptions.

Interactive learning sessions were portrayed as dynamic environments where learning becomes a shared experience, contributing to a sense of collective purpose and shared goals. Participants highlighted the opportunity to collaborate with peers, which not only facilitates a deeper understanding of the topic but also fosters a sense of achievement as a team. The interactive learning experience within a team-based environment not only enhances understanding of course material but also cultivates essential teamwork skills that are invaluable for future professional endeavors. Together, this painted a comprehensive picture of the multifaceted nature of effective teamwork, providing a roadmap for team-based learning seeking to optimize the collaborative potential. The appreciation of students to understand the concept via interactive sessions and the reluctance to participate are depicted.

## Discussion

This study provides valuable insights into the effectiveness of collaborative learning approaches among undergraduate medical students. The findings indicated a generally positive response from the participants, with a majority strongly agreeing with statements related to contributing ideas, listening to classmates, being prepared for work, and supporting group members. This suggests a high level of engagement and cooperation within the TBL sessions, highlighting the benefits of collaborative learning in enhancing teamwork skills and knowledge sharing [15,16].

The themes identified in our study were conceptual clarity, collaborative learning culture, communication skills, informative team discussions, and interactive learning for team development. These themes underscore the importance of effective communication, knowledge sharing, and interactive learning experiences in fostering team unity and enhancing individual skills. Another exploratory study was conducted on teaching and learning strategies at a tertiary care hospital in Pakistan [17]. Mansoor et al. [18] conducted a study on the comparative evaluation of academic performance in second-year MBBS students between those taught by TBL versus SGD (small group discussion); however, both were found to be equally effective. Another study showed that implementation of TBL increased students' responsibility for their learning and helped the students bridge the gap in their cognitive knowledge, which resulted in increased scores in the summative assessment of students who undertook TBL sessions [19]. A study by Woodcock et al., [20] concluded that TBL stimulates critical thinking amongst the students and makes them realize how clinicians think and apply basic sciences content in the clinical setup. Another study revealed that critical thinking dimensions such as inquisitiveness and analyticity are statistically significant [21]. TBL enhances students' problem-solving skills by allowing them to gather relevant material, debate, ask questions, and receive feedback. It also boosts confidence, enhances study habits, and promotes responsibility in education [22]. Interactive learning sessions, facilitated by clear goals, effective communication, and mutual respect, are appreciated for their informative nature, team development, improved memorization, and time-saving benefits [23,24]. Students take a proactive approach to problem-solving by actively participating in class discussions. This process of mutual learning emphasizes how new information can be learned from peers. When working in groups, students actively help classmates grasp difficult subjects, showcasing their strongest skills, as shown by these studies [25,26].

Good communication skills are essential for good teamwork. They convert abstract ideas into workable plans. The study shows that team-based learning facilitates the improvement of communication skills among participants. These help participants become more successful and have a deeper grasp of the difficult topics [27].

Interactive training sessions are an effective way to build a team by strengthening individual members and promoting togetherness. These group activities foster dynamic settings where education becomes a shared experience, strengthening the sense of purpose and objectives among participants. Working together with peers promotes team success and greater understanding. Effective interactive sessions are an important tool for learning and teamwork since they help with memorization and clarify ambiguity about particular topics [8,27].

Teamwork enhances course comprehension, develops collaboration skills, fosters rapport, and is fulfilling. Theme analysis highlights connections, communication, debates, and interactive learning for maximum potential.

The study also has some limitations, such as potential selection bias from a single institution, limited generalizability due to geographical variation, self-reporting bias in data collection, lack of longitudinal data for long-term assessment, and methodological challenges in data analysis. Feedback from faculty was not considered.

## Conclusions

The combined findings from both quantitative and qualitative analyses provide a nuanced understanding of collaborative learning in TBL. By contributing to the enhancement of TBL practices in medical education, this research offers valuable implications for educators looking to incorporate these learning approaches into their teaching strategies. Overall, the study highlights the positive effects of TBL in promoting peer-to-peer engagement, active engagement, teamwork skills, and knowledge sharing among undergraduate medical students, ultimately contributing to a more enriching and effective learning experience.

This study promotes the use of team-based learning (TBL) in developing countries, highlighting the need for resources, training, and hands-on workshops for faculty members to design and facilitate TBL activities effectively. It aims to optimize student engagement and learning outcomes.
